# A HuR/TGF-β1 feedback circuit regulates airway remodeling in airway smooth muscle cells

**DOI:** 10.1186/s12931-016-0437-1

**Published:** 2016-09-22

**Authors:** Na Wang, Di Yan, Yi Liu, Yao Liu, Xianmin Gu, Jian Sun, Fei Long, Shujuan Jiang

**Affiliations:** Department of Pulmonary Medicine, Shandong Provincial Hospital Affiliated to Shandong University, Jinan, 250021 China

**Keywords:** Asthma, Human antigen R, Transforming growth factor, α-smooth muscle actins, Collagen 1a, Airway remodeling, Post-transcription regulation

## Abstract

**Background:**

Asthma is a worldwide health burden with an alarming prevalence. For years, asthma-associated airway injury remains elusive. Transforming growth factor β1 (TGF-β1) is a pleiotropic cytokine that has been shown to be involved in the synthesis of the matrix molecules associated with airway remodeling. Human antigen R (HuR), the member of the Hu RNA-binding protein family, can bind to a subset of short-lived mRNAs in their 3′ untranslated regions (UTR). However, the functional roles and relevant signaling pathways of HuR in airway remodeling have not been well illustrated. Thus, we aim to explore the relationship between HuR and TGF-β1 in platelet derived growth factor(PDGF)-induced airway smooth muscle (ASM) cells and asthmatic animal.

**Methods:**

Cultured human ASM cells were stimulated by PDGF for 0, 6, 12 and 24 h. Western blotting, RT-PCR and immunofluoresence were used to detect the expression of HuR, TGF-β1, α-smooth muscle actins (α-SMA) and collagen type I (Col-I). Then knockdown of HuR, flow cytomerty was used to detect the morphological change and western blotting for functionally change of ASM cells. Furthermore, the interference of TGF-β1 and exogenous TGF-β1 were implemented to testify the influence on HuR. A murine OVA-driven allergic model based on sensitization and challenge was developed. The inflammatory response was measured by bronchoalveolar lavage fluid (BALF), airway damage was analyzed by hematoxylin and eosin staining, airway remodeling was assessed by sirius red staining and periodic acid-schiff staining, the expression level of HuR, TGF-β1 and α-SMA were measured by RT-PCR, western blotting and immunohistochemistry.

**Results:**

Here, we found that PDGF elevated HuR expression both at mRNA and protein level in cultured ASM cells at a time-dependent manner, which was simultaneously accompanied by the enhanced expression of TGF-β1, α-SMA and Col-I. Further study revealed that the knockdown of HuR significantly increased the apoptosis of ASM cells and dampened TGF-β1, Col-I and α-SMA expression. However, interfering TGF-β1 with siRNA or extra addition of TGF-β1, HuR could restore its production as well as Col-I. Compared with normal mice stimulating with PBS, OVA-induced mice owned high amount of inflammatory cells, such as eosinophils, lymphocytes and neutrophils except macrophages. HE staining showed accumulation of inflammatory cells surrounding bronchiole and sirius red staining distinguished collagen type I and III deposition around the bronchiole. Higher abundance of HuR, TGF-β1 and α-SMA were verified in OVA-induced mice than PBS-induced mice by RT-PCR, western blotting and immunohistochemistry.

**Conclusions:**

A HuR/TGF-β1 feedback circuit was established to regulate airway remodeling in vivo and in vitro and targeting this feedback has considerable potential for the intervention of asthma.

## Background

Airway remodeling, a fundamental pathogenic feature of asthma, is characterized by matrix deposition and enhanced smooth muscle mass in the airways. For patients with recurring episodes of asthma, structural changes are one of the most important reasons for the deterioration of lung function, which is now becoming a life-threatening challenge in the treatment of asthma [1, 2]. Grainge [3] had confirmed that collagen deposition and goblet cell hyperplasia contributed to airway remodeling in mild asthma, but detailed mechanism remained to be elucidated.

TGF-β1, a pleiotropic cytokine that had been evidenced to be involved in the synthesis of matrix molecules in the ASM cells, especially on the synthesis of collagen types I, III, IV, VII and X, fibronectin and proteoglycans [4], has been implicated in the pathogenesis of airway remodeling in asthma [5–7]. For years, the mechanisms underlying the development of fibrosis have been extensively studied and multiple signal pathways are involved in, such as the integrin α5β6 [8], phosphatidylcholine-specific phospholipase C, protein kinase C-delta [9], the integrin receptor α5β1 [10], the canonical Smad-dependent signaling pathway [11] and p38 MAPK [12]. In recent years, a series of observations reported that Angiotensin II could be a putative mediator in increasing TGF-β1 and Col-I deposition [13–15]. Although the roles of these signaling pathways have been well established by ample experimental studies, no specific inhibitor applicable in asthma has been described.

HuR, the sole ubiquitous member of the Hu RNA-binding protein family, can bind to a subset of short-lived mRNAs that harbor AU-rich elements (AREs) in their 3′ untranslated regions (UTR), which is called post-transcriptional gene regulation that coordinating the process of mRNA splicing, transport, turnover, and translation in multiple development processes and diseases [16]. These mRNAs include c-fos, VEGF, TNF, α,β-catenin, c-myc, cyclooxygenase 2, myogenin, MyoD, and granulocyte/macrophage colony-stimulating factor (GM-CSF) [17–19]. Fan [20] has showed that HuR critically regulates the epithelial response by associating with multiple functionally related ARE-bearing inflammatory transcripts, and Zhang [21] also reported that HuR participated in ASM proliferation by mediating CyclinD1 expression. In particular, TGF-β1 3′UTR was reported to be a putative target of HuR in human cancer cells [22]. However, the underlying relationship between HuR and TGF-β1 in regarding to airway remodeling has been reported in few studies. So it is still a challenge to explore the possible pathogenic mechanisms of refractory airway remodeling.

In our study, we found a novel HuR/TGF-β1 feedback circuit that modulating airway remodeling in airway smooth muscle cells and in asthmatic mouse firstly. In vitro, we detected that HuR and TGF-β1 demonstrated high expression in a time-dependent manner under the stimulation of PDGF, a strong stimulus for asthmatic response. Besides, α-SMA and Col-I simultaneously exhibited over-expression. Furthermore, knockdown of HuR led to an increase of ASM cells apoptosis and down-regulation of TGF-β1, α-SMA and Col-I. Moreover, the half-life of TGF-β1 was shorter compared with the control. However, interfering TGF-β1 with siRNA can obviously decrease HuR and Col-I expression. But exogenous TGF-β1 could recover HuR and Col-I expression. In vivo, OVA-induced mice showed widely infiltration of inflammatory cells surrounding the bronchioles in comparison with PBS-induced mice. Sirius red staining distinguished higher deposition of collagen type I and III around the bronchiole in OVA-induced mice then in PBS-induced mice. RT-PCR, western blotting and immunohistochemistry all showed higher levels of HuR, TGF-β1 and α-SMA in OVA -induced mice than PBS-induced mice. Thus we hypothesized that a HuR/TGF-β1 feedback is involved in airway remodeling and targeting them might have considerable potential for the control of asthma.

## Methods

### Cell culture and animals

Human bronchial smooth muscle (ASM) cells were purchased from Cambrex Bio Science (Walkersville, MD). Cells were cultured in DMEM cell medium (Hyclone) containing 10 % fetal bovine serum supplemented with 100 U/ml penicillin and 100 μg/ml streptomycin. Cells at passages between 4 and 11 were used for all experiments, because later passage cells showed greater interculture variability. For PDGF treatment, cells were plated and cultured in complete DMEM overnight. Next day, cells were divided into different groups according to the different compounds added. For control group, no compounds were administrated. For PDGF group, cells were stimulated with 20 ng/ml PDGF and cultured for 0, 6, 12 and 24 h.

**Fig. 1:**
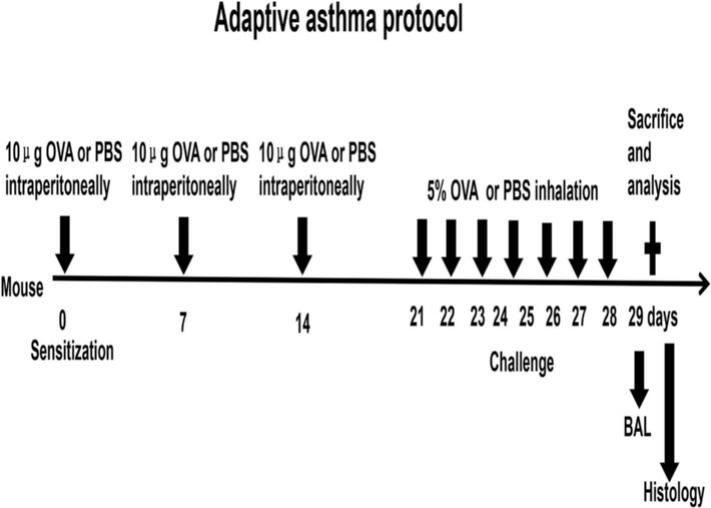
Experimental protocols for chronic OVA-induced asthma in mice. BALB/c mice received three intraperitoneal sensitizations with ovalbmin grade V mixed with the adjuvant aluminium hydroxide for emulsified on days 0, 7 and 14, followed by seven consecutive challenge each day with 5 % ovalbumin grade III aerosols for 30 min. The negative control mice were received the same volume of phosphate-buffered saline (PBS) by intraperitoneal injection for three times, then undergo airway challenge. No exogenous adjuvant was given at any time. The mice were sacrificed with an over dose anaesthetized, BALF was performed, and the lungs were dissected for histological analysis

A total of 30 BALB/c female mice (age, 8–10 weeks; weight, 22 ± 2 g) were purchased from the Laboratory Animal Center of Shandong University and were housed under specific pathogen-free conditions. All mice had access to food and water free. Briefly, a model consists of three intraperitoneal sensitization on days 0, 7 and 14, followed by seven consecutive challenges with 5 % ovalbumin grade III (Sigma-Aldrich) aerosols for 30 min each day. For sensitization, OVA grade V was dissolved to a final concentration of 20 μg/ml in 500 μl sterile PBS per mouse. Add alum to a concentration of 2 mg/ml. The negative control mice received the same volume of phosphate-buffered saline (PBS) by intraperitoneal injection for three times, then undergo airway challenge. No exogenous adjuvants were given at any time [23]. More detailed protocol was listed in Fig 1. All experimental procedures of the mice were performed in compliance with the guidelines of the Institutional Animal Care and Use Committee. The protocol was approved by the Committee on the Ethics of Animal Experiments of Shandong Provincial Hospital affiliated to Shandong University.

## Materials

Anti-HuR (ab17397, Abcam), TGF-β1 (ab92486,Abcam), α-SMA (ab5694, Abcam), collagen I (ab34710, Abam), and actin (ab8226, Abcam) antibodies were available for western blotting, immunohistochemistry and immunofluoresence. Reverse kit was purchased from Takara (Japan). PDGF-BB was purchased from Peprotech and recombinant human TGF-β1 was bought from Invitrogen. Small interference RNA duplexes against HuR and TGF-β1 plus negative control were synthesized in Genepharm (Shanghai, China). Lip3000 and Trizol was purchased from Invitrogen. TGF-β1 and human Col-I ELISA kit were bought from Bioworld. All other reagents were enrolled from domestic companies with good reputations unless specifically stated.

### Real-time PCR

Cells were harvested and total RNA was isolated with Trizol. In mice, the total RNA was extracted from the right lungs using Trizol (Invitrogen, USA). Reverse transcription of 1 μg RNA was carried out according to the instructions of Takara RT kit (Japan). Quantitect SYBR Green kit (USA) was used for amplification and fluorescence was detected by using ABI Prism 7700 Detection System. GAPDH was run as internal control and 2^−ΔΔCt^ method was performed for analysis (n = 3). To test the TGF-β1 mRNA stability, ASM cells transfected with ConsiRNA or HuRsiRNA were further treated with PDGF or without for 6 h. Then actinomycinD (5 μg/ml) was added in every well immediately for 0, 4, 8 and 12 h and RNA was extracted at indicated time to examine the RNA abundance for stability analysis. Primers sequences were listed at Table 1.

**Table 1:** Primers and sequences used in this study

Name	Sequence
GAPDH real time primer	F-5′-GCAAGTTCAACGGCACAG-3′
	R-5′-GCCAGTAGACTCCACGACATA-3′
HuR real time primer	F-5′-GGCGAGCATACGACA3′
	R-5′-TATTCGGGATAAAGTAGC3′
TGF-β1 real time primer	F-5′-CCCACTGATACGCCTGAG-3′
	R-5′-TGAAGCGAAAGCCCTGTA-3′
Col-I real time primer	F-5′-CACTCAGCCCTCTGTGCCT-3′
	R-5′-ACCTTCGCTTCCATACTCG-3′
ConsiRNA	F-5′-UUCUCCGAACGUGUCACGUTT-3′
	R-5′-ACGUGACACGUUCGGAGAATT-3′
HuRsiRNA	F-5′-CAACAAGUCCCACAAAUAAUU-3′
	R-5′-AAUUAUUUGUGGGACUUGUUG-3′

### Western blotting

Harvested cells were rinased with PBS and lysed by RIPA containing 50 mM Tris, 150 mM NaCl, 1 % Triton X-100, 1 % Sodium deoxycholate, 0.1 % SDS plus protease inhibitor PMSF. Lung tissues were homogenized with ice-cold RIPA plus PMSF. Protein concentration was tested by the BCA protein assay kit. Protein samples (30 μg per lane) were submitted to electrophoresis on 10 % SDS-polyacrylamide gel and resolved to PVDF membrane. After blocking for 1 h (5 % non-fat milk in Tris buffered saline plus 0.1 % Tween 20), the membranes were incubated in blocking buffer at 4 °C overnight with anti-HuR (1:2000), anti-TGF-β1 (1:1000), anti-α-SMA (1:150) and anti-Col I (1:1000). After three times washing next day, the membranes were probed with HRP (1:5000 in blocking buffer) linked secondary antibodies, and visualized with ECL reagent (Thermo Scientific).

### Immunofluorescence

After the ASM cells were stimulated with PDGF for 0, 6, 12 and 24 h respectively, cells were fixed in 4 % paraformaldehyde for 15 min and permeabilized for 20 min in phosphate-buffered saline containing 0.5 % Triton X-100. After incubation in blocking buffer (goat serum) for 1 h at 37 °C, cover slips were incubated in a 1:50 dilution of anti-TGF-β1 and anti-HuR prepared in blocking buffer overnight. Cover slips were washed with blocking buffer next day and incubated for at least 1 h with TRITC-labeled goats anti-rabbit IgG or anti-mouse IgG. Then cover slips were washed for thee times with blocking buffer and cells were dyed nucleus with DAPI for 15 min. After carefully washing, cell images were acquired with a fluorescence inverted microscope (Olympus BX50).

### RNA-interference

According to the manufacturer’s instructions, the trypsinized ASM cells were resuspended at a density of (0.25-1) × 10^6^/ml in 6-well plate. 5 μl Lip3000 was diluted in 125 μl OPTI-MEM and 2.5 μg DNA plus 5 μl P3000 were diluted in 125 μl OPTI-MEM. Then two compounds were well mixed and incubated 10 min at room temperature to form transfected complexes. Then total 250 μl complexes were dispensed into a culture plate containing 1750 μl complete DMEM medium and mixed with the cell suspension gently. Additional experiments were performed after transfection for 48 h.

### Flow cytometry and CCK8 assay

Apoptosis assay: ASM cells were divided into Consi, Consi + PDGF, HuRsi and HuRsi + PDGF groups. ASM cells were then harvested, washed and resuspended with PBS. Apoptotic cells were determined with an Alexa Fluor 488 Annexin V/Dead Cell Apoptosis kit (Invitrogen) according to the manufacturer’s protocol. Briefly, the cells were washed and subsequently incubated in 100 μl of 1X Annexin binding buffer containing 5 μl of Annexin V-FITC and 2 μl of propidium iodide (PI) for 15 min in the dark. Then, apoptosis was analyzed using a FACScan laser flow cytometer. Data were analyzed by using FlowJo software.

Cell proliferation assay: ASM cells were divided into Consi, Consi + PDGF, TGFsi, TGFsi + PDGF groups. 10 μl CCK8 was added to each well. Cells were further cultured for 1 h, and then when each well turned to orange, the optical density(OD) was measured at 450 nm using a multiscan reader. The average OD of four wells for each group was calculated.

### ELISA

The concentration of TGF-β1 and Col-I in the cultured serum was measured by ELISA-kits. The protocols were followed according to the manufacturer’s instructions. Briefly, the cultured serum samples collecting from the Consi and HuRsi group after cultured for indicated time were added in triplicate to 96-well plates with 100 μl per well. The appropriate biotinconjugated antibodies were added to each well. The samples were incubated at room temperature for 2 h. The wells were then aspirated, and each well was washed five times. The substrate solutions were added to each well, and were incubated for 30 min at room temperature in the dark. The optical density (OD) of each well was determined using a microplate reader that was set to 450 nm. A standard curve was created of the average of the OD duplicate readings. Data was analyzed by CurveExpert 1.3 and SPSS 19.0.

### Bronchoalveolar lavage fluid (BALF) and cell collection

All mice were sacrificed within 24 h after the last treatment. The left major bronchus was tied with a string, which was inserted with a 24-gauge needle, and the BALF was obtained by the infusion and collection of 0.5 ml of saline. The infusion and collection steps were repeated for 3 times. The BALF was centrifuged at 2000 rpm for 10 min at 4 °C.The different cell counts in the BALF were carried out as described [24]. In brief, the pellet was resuspended with 0.5 ml of saline and the different cell counts were performed with Giemsa staining. The different cell counts were determined by light microscopy from a count of at least 400 cells. The percentages of macrophages, eosinophils, lymphocytes and neutrophils were determined by counting their numbers in randomly selected high-power fields and by dividing this number by the total number of cells per high-power field. All of the counts were performed by the same observer in a blinded manner and in a randomized order at the end of the study.

### Histology

The lungs were dissected from the chest cavity after the lavage. The left lungs were immediately fixed in 4 % paraformaldehyde and paraffin-embedded, and tissue sections (5 mm) were prepared. To assess airway remodeling, the sections were stained with periodic acid Schiff stain (PAS) for goblet cells and with Sirius red staining for collagen deposition, as described previously [25]. Briefly, goblet cell upregulation within the airway epithelia was assessed by measuring the length of the airway basement membrane that was covered by goblet cells. Peribronchial collagen thickness was measured using Image-Pro Plus software (version 6, Media Cybernetics, USA). Three bronchioles were selected at randomly from each section and the mean depth of collagen in the basement membrane was determined from five measurements around the bronchiole. For immunohistochemical analysis, the sections were initially incubated with anti-α-SMA rabbit monoclonal antibody (1:150; Abcam), anti-HuR rabbit monoclonal antibody (1:500; Abcam) and anti-TGF-β1 rabbit polyclonal antibody (1:100; Abcam) at 4 °C overnight, then were incubated with HRP-conjugated goat anti-rabbit (1:50; Abcam) for 30 min at 37 °C. Positive staining was detected with HRP-conjugated streptavidin, visualized with 3,3′-diaminobenzidine and counterstained with hematoxylin [26]. Finally, the sections were mounted, cover-slipped, and examined under a light microscope (Olympus BX50). The extent of positive area were analyzed by using Image-Pro Plus 4.5.

### Statistical analysis

All results were performed at least three independent experiments. All data were processed by SPSS version 19.0 and quantitative data were shown as mean ± standard deviation (SD). A student’s t test (two-tailed) was used to compare two groups and One-way ANOVA for multiple comparisons. Quantity one V4.62 and Graphpad Prism 5.0 were used to quantify relative expression of proteins. Values of *P* < 0.05 were considered statistically significant.

## Results

### PDGF treatment promoted TGF-β1 along with Col-I and α-SMA expression

**Fig. 2:**
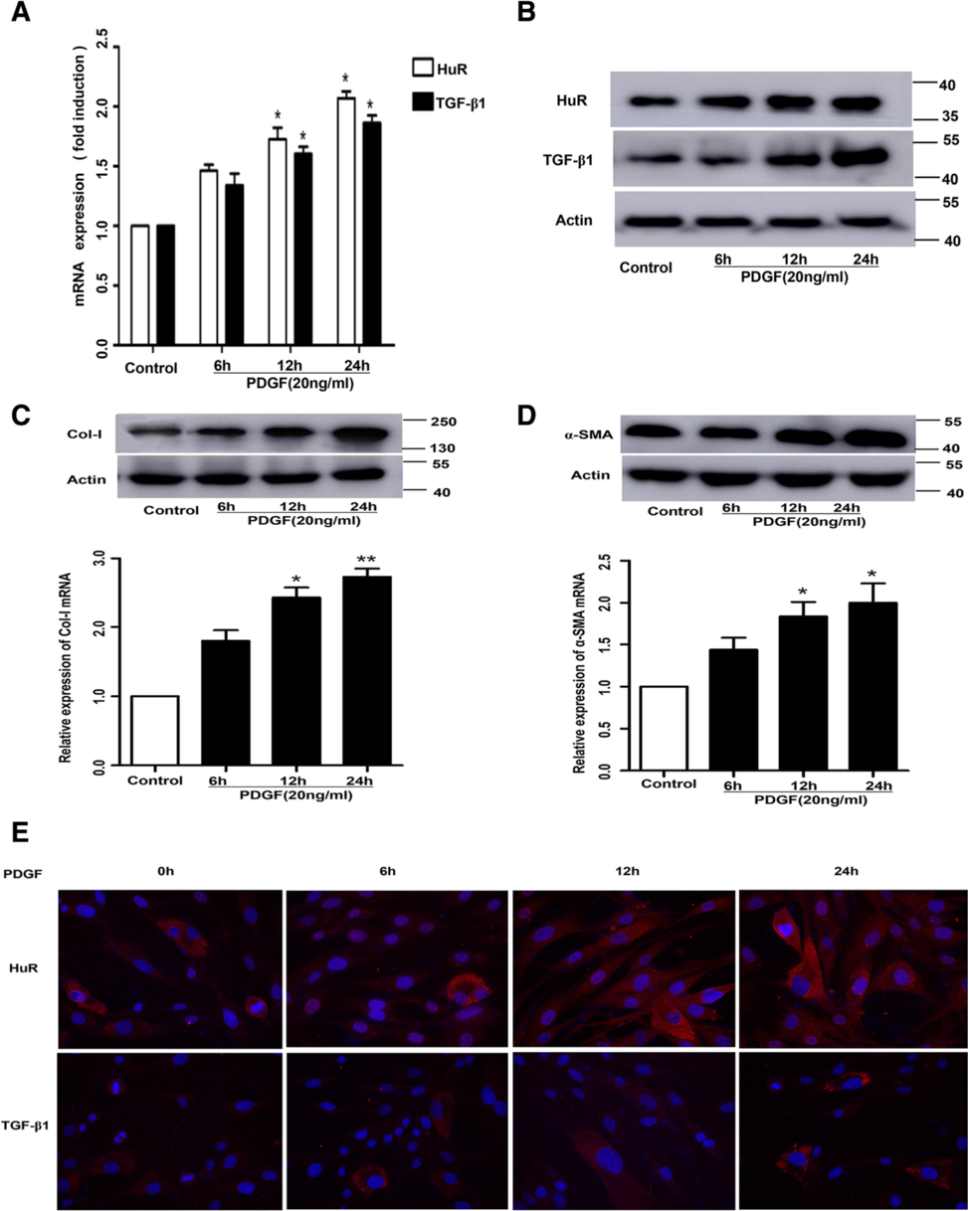
PDGF treatment promoted TGF-β1 along with Col-I and α-SMA expression. **a**–**d** HuR, TGF-β1, Col-I and α-SMA mRNA levels were measured by RT-PCR and protein levels were measured by western blotting in ASM cells under the stimulation of PDGF for various durations (0, 6, 12, 24 h). GAPDH in RT-PCR and β-actin in western blotting were the internal control. Density ratio of bands in western blotting was calculated and represented as relative expression level. Each value represents the mean ± SD of three independent experiments. **e** Immunofluorescence analysis was used to detect subcellular distribution of HuR and TGF-β1 in ASM cells following the same treatment. *P < 0.05 compared with control; **P < 0.01 compared with control

In our present study, we found that HuR expression elevated in a time-dependent manner under the stimulation of PDGF in cultured ASM cells, which was consistent with the previous study (Fig 1a&b). To investigate whether PDGF treatment could also affect the expression of TGF-β1 plus Col-I and α-SMA, cultured ASM cells were treated with 20 ng/ml PDGF for 0, 6, 12 and 24 h. TGF-β1 expression was detected at both the mRNA and protein levels using real-time PCR and western blotting, respectively. Compared with control (without PDGF treatment), PDGF treatment for 6, 12 and 24 h elevated TGF-β1 mRNA levels by 27, 60 and 87 % separately(*P* < 0.05) (Fig. 2a). Similar alterations could also be shown at TGF-β1 protein levels, evidenced by 31, 69 and 106 % elevation (*P* < 0.05) (Fig. 2b). Likewise, Col-I and α-SMA, a marker of smooth muscle cells, were also increased in a time-dependent manner (both *P* < 0.05) (Fig 2c&d). The relative contents of all above three proteins were demonstrated in Table 2. The subcellular distributions of HuR and TGF-β1 showed that PDGF stimulation for 6 h, especially in 12 h, induced a significant elevation of total nuclear or cytoplasmic HuR and TGF-β1 abundance (Fig 2e). Collectively, these results above provided direct evidence that PDGF treatment promoted TGF-β1 along with Col-I and α-SMA expression.

**Table 2:** Relative expression of TGF-β1, Col-I and α-SMA in mRNA and protein levels

Group	TGF-β1		Col-I		α-SMA	
	mRNA	Protein	mRNA	Protein	mRNA	Protein
0 h	1.00 ± 0.00	0.67 ± 0.08	1.00 ± 0.00	0.89 ± 0.10	1.00 ± 0.00	0.88 ± 0.08
6 h	1.27 ± 0.06	0.88 ± 0.10^a^	1.12 ± 0.14	1.02 ± 0.07	1.35 ± 0.14	1.22 ± 0.17^a^
12 h	1.60 ± 0.10^b^	1.13 ± 0.10^ab^	1.48 ± 0.17^b^	1.13 ± 0.08^b^	1.73 ± 0.17^b^	1.61 ± 0.14^ab^
24 h	1.87 ± 0.10^bc^	1.38 ± 0.10^abc^	1.69 ± 0.10^bc^	1.33 ± 0.10^bc^	2.07 ± 0.10^bc^	1.98 ± 0.15^abc^

Note:compared with 0 h, ^a^ *P* < 0.05;compared with 6 h, ^b^ *P* < 0.05; compared with 12 h, ^c^ *P* < 0.05

### HuR silencing increased the apoptosis of ASM cells

**Fig. 3:**
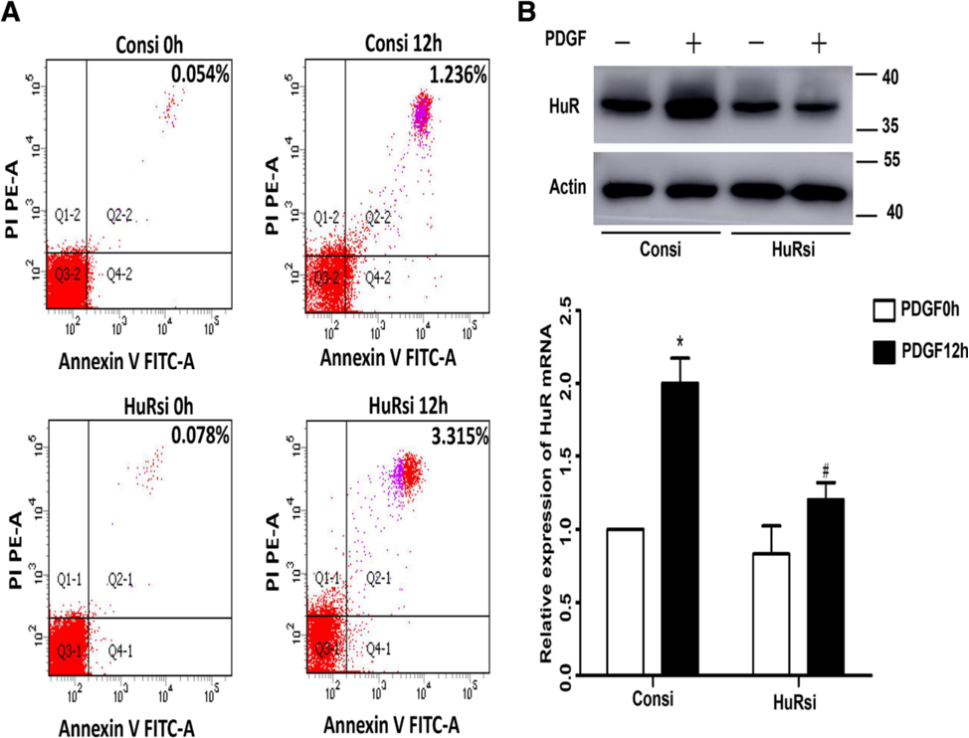
HuR silencing changed the function of ASM cells. **a** FACS analysis of the apoptotic and necrotic cells using Annexin V/PI after cultured for 0 h or 12 h in the Consi and HuRsi groups. Annexin V/PI staining with FACScan dot plot analysis were used to divide the treated and control cells into three groups: living cells (lower left quadrant), necrotic cells (upper right quadrant). **b** Knockdown efficiency of HuR was analyzed by RT-PCR and western blot assay. GAPDH in RT-PCR and β-actin in western blotting were the internal control. Density ratio of bands in western blotting was calculated and represented as relative expression level. *P < 0.05 compared with control; #*P* < 0.05 control siRNA vs. HuR siRNA upon the 12 h stimulation of PDGF

Annexin V was used to detect the effects of HuR silencing on the morphological change of ASM cells. As shown in Fig 3a, there were no differences between the Consi and HuRsi group at the beginning. However, we could see the proportion of apoptosis in the Consi group after cultured for 12 h were 1.236 %, while in the HuRsi group was 3.315 % (*P* < 0.05). Western blotting was better to evaluate the functional change after knockdown. As seen from Fig 3b, the relative expression of HuR protein in the HuRsi group was reduced 44.1 % compared with the Consi group. mRNA levels showed that a 28.8 % difference between the Consi and HuRsi groupwas found. The results above showed that HuR could protect ASM cells from apoptosis to a certain extent.

### HuR silencing decreased TGF-β1 expression via decreasing the mRNA stability

**Fig. 4:**
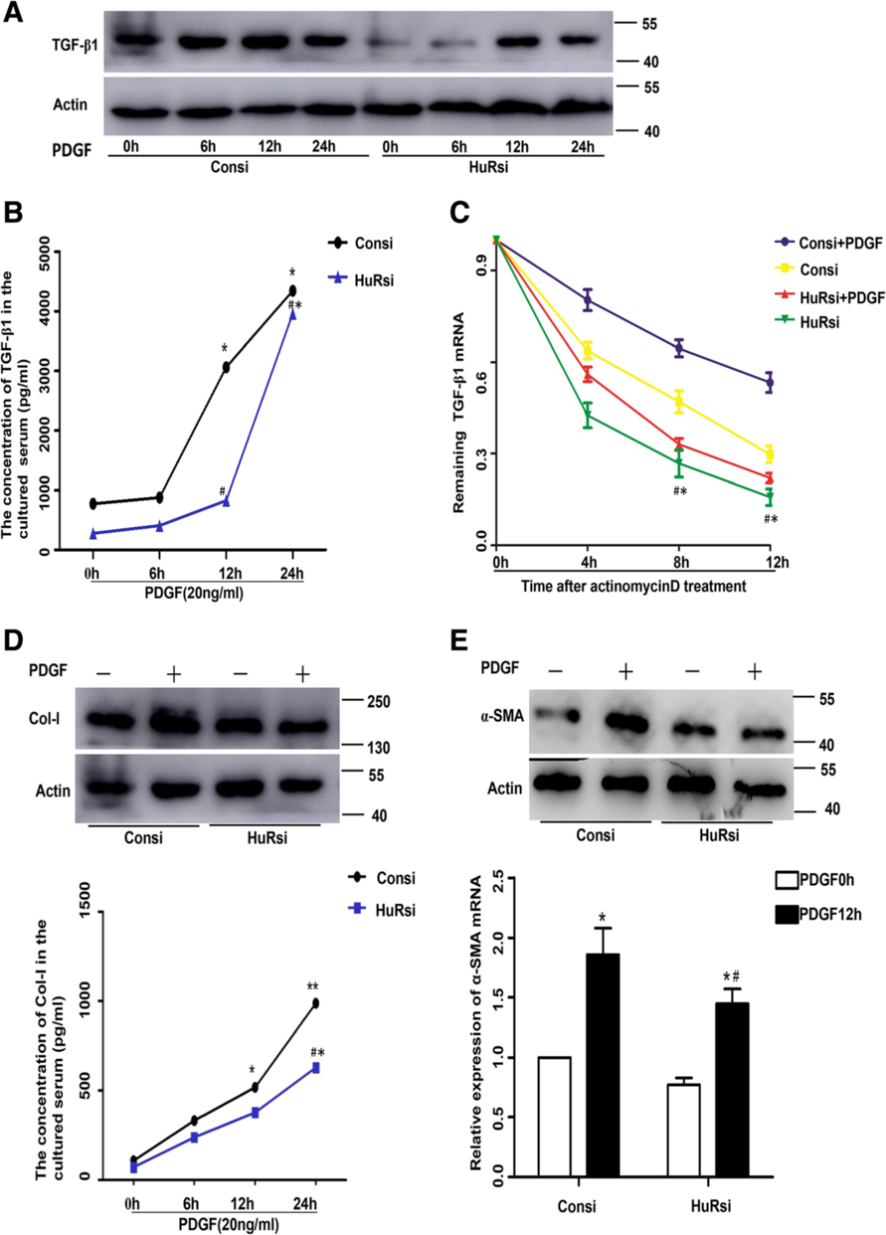
HuR silencing attenuated TGF-β1 along with Col-I and α-SMA expression and decreased the mRNA stability of TGF-β1. **a** Silenced HuR downregulated endogenous TGF-β1 protein expression at a time-dependent manner. Density of each band was quantified using Image software and the ratio of bands was calculated and represented as relative expression level. **b** The concentration of TGF-β1 in the cultured serum was detected by ELISA kits. Data was analysed by Curve Expert and showed by mean ± SD. **c** Remaining TGF-β1 mRNA were detected in siHuR or Consi group with stimulation of actinomycinD for 0, 4, 8,12 h. RNA expression was 50 % of the initial level [t1/2 = Ln(0.5)/slope]) representing the half-life. Graphpad prism5.0 was used to analyse data and data was shown with mean ± SD. **d** Western blotting showed significant decrease of Col-I after the knockdown of HuR. The outcomes of the concentration of Col-I in the cultured serum was in consist with western blotting. **e** Western blotting demonstrated alleviatin of α-SMA expression after silencing HuR. *P < 0.05 compared with control; #*P* < 0.05 control siRNA vs. HuR siRNA upon the stimulation of PDGF for 12 h or 24 h

We employed small interference RNA to determine whether suppressing HuR expression could decrease the PDGF-induced TGF-β1 expression in ASM cells. Western blotting analysis demonstrated that HuR silencing decreased HuR expression to very low level, simultaneously the TGF-β1 protein content was also decreased in the HuR-silenced group (HuRsi) compared with the silencing control groups (Consi) in a time-dependent manner (Fig 4a). The concentration of TGF-β1 in the cultured serum also showed the same alteration that TGF-β1 obviously elevated in the Consi group especially in 12 h compared with the HuRsi group (Fig 4b) (Table 3).

**Table 3:** The concentration of TGF-β1 and Col-I in the cultured serum (pg/ml)

Group	TGF-β1		Col-I	
	Consi	HuRsi	Consi	HuRsi
0 h	773.33 ± 16.32	277.33 ± 9.93	106.60 ± 5.19	70.95 ± 4.90
6 h	877.97 ± 16.03	407.77 ± 7.14^a^	332.11 ± 10.91	238.5 ± 9.28
12 h	3060.34 ± 82.53	828.05 ± 11.67^ab^	518.23 ± 14.62	376.50 ± 12.44^b^
24 h	4338.13 ± 72.48	3958.06 ± 80.98^abc^	988.56 ± 20.11	628.51 ± 18.91^bc^

Note:compared with 0 h, ^a^ *P* < 0.05;compared with 6 h, ^b^ *P* < 0.05; compared with 12 h, ^c^ *P* < 0.05

On the basis of the observations that PDGF treatment enhanced the TGF-β1 protein level and the expression of TGF-β1 was decreased after the knockdown of HuR. We further investigated whether mRNA stability change occurred in the transfected ASM cells. The ASM cells were left untreated or stimulated with PDGF for 6 h, then either harvested or cultured in the presence of the transcriptional inhibitor actinomycinD (5 μg/ml) for an additional 4 h, 8 h and 12 h. As shown in Fig 4c, treatment with actinomycinD revealed that there was a tiny but consistent decrease in TGF-β1 mRNA half-life (the time expressed in hours at which mRNA expression was 50 % of the initial level) in the HuRsi group. However, the half-life in the Consi group was significantly prolonged especially under the PDGF stimulation, indicating that HuR might enhance the TGF-β1 mRNA stability. Moreover, Bai [27] ever demonstrated direct interaction between HuR and TGF-β1 3′UTR by performing RNA-IP in cardiac fibroblasts. Thus, we considered that HuR mediated TGF-β1 expression via stablizing TGF-β1 mRNA in ASM cells.

Furthermore, the western blotting and the cultured medium showed lower expression of Col-I in the HuRsi group than in the Consi group (Fig 4d) (Table 3). Likewise, the expression of α-SMA in the HuRi group were reduced a lot especially in 12 h (Fig 4e). These results facilitated the hypothesis that HuR might effectively modulate the expression of Col-I and α-SMA via modulating the expression of TGF-β1.

### Suppression of TGF-β1 ablates HuR and Col-I expression but exogenous TGF-β1 restored HuR and Col-I expression in ASM cells

**Fig. 5:**
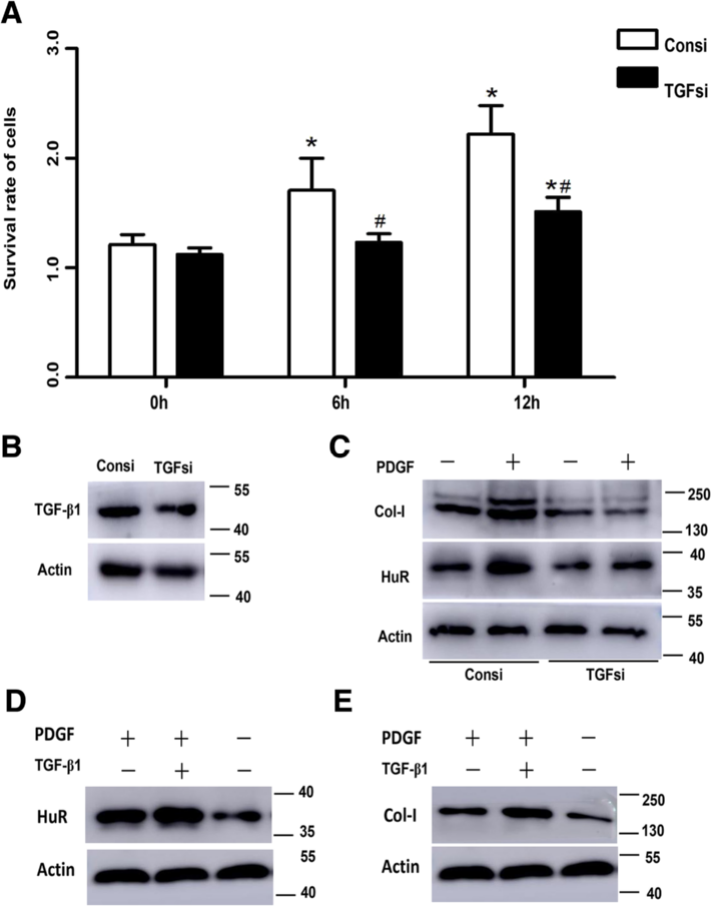
Suppression of TGF-β1 ablated HuR and Col-I expression but exogenous TGF-β1 restored HuR and Col-I expression. **a** Knockdown of TGF-β1 decreased ASM cells viability by CCK8 assay. **b** Small interfere RNA decreased TGF-β1 expression compared with control siRNA. (**c**) After silencing TGF-β1, HuR and Col-I were also alleviated even though under the stimulation of PDGF. **d**–**e** Exogenous TGF-β1 treatment increased endogenous HuR and Col-I expression.β-actin in western blotting were the internal control. Density ratio of bands in western blotting was calculated and represented as relative expression level. *P < 0.05 compared with control; ^#^ *P* < 0.05 control siRNA vs. TGF-β1 siRNA

In order to test whether TGF-β1 could affect HuR expression, ASM cells were transfected with small interference RNA to alleviate TGF-β1 expression. We found that ASM cells in TGFsi group owned lower survival rate than the Consi group on the PDGF stimulation for 12 h (Fig 5a). As seen from Fig 5b, the relative expression of TGF-β1 protein in TGFsi group was reduced 23.8 % compared with the Consi group. Upon the affection of TGF-β1 on HuR and Col-I, the grey value of bands alleviated respectively (Fig 5c). However, when the TGF-β1-blockaded ASM cells were treated with 4 ng/ml TGF-β1 for 12 h, HuR elevated 89 % under the stimulation of extra TGF-β1 and PDGF compared with the control group, while PDGF treatment alone elevated 40 % expression of HuR (Fig 5d). Besides, TGF-β1 treatment could also improve Col-I expression (Fig 5e). These data further corroborated our hypothesis that a HuR/TGF-β1 feedback existed among ASM cells to regulate airway remodeling, especially in the expression of Col-I and α-SMA.

### OVA-induced mice owned high amount of inflammatory cells and collagen type I, III and IV deposition

**Fig. 6:**
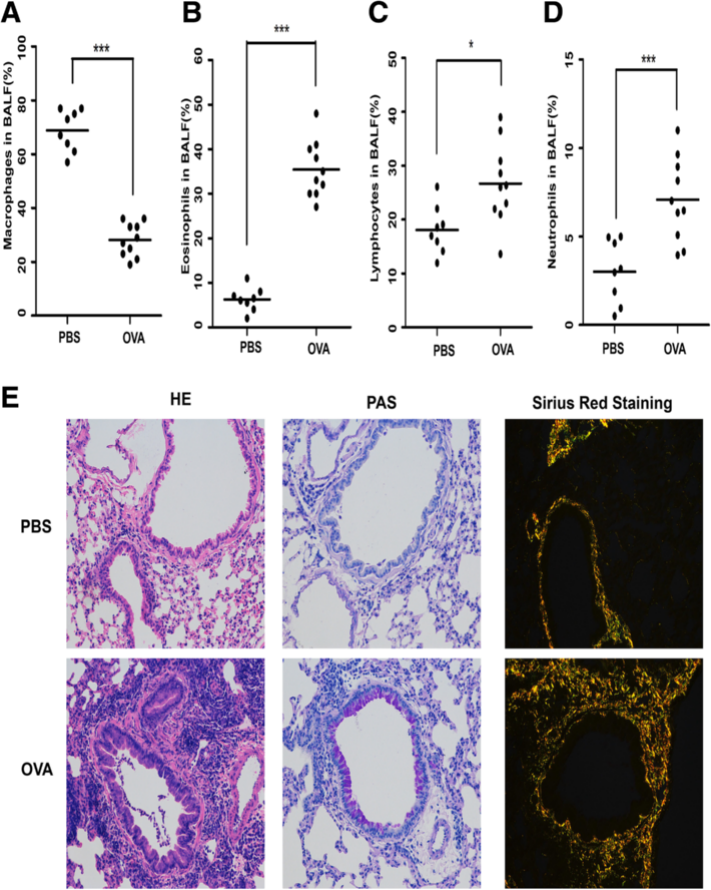
OVA-induced mice owned high amount of inflammatory cells and type I and III collagen deposition. Inflammatory cell recruitment in BALF of OVA-induced asthma: **a** Macrophages; **b** Eosinophils; **c** Lymphocytes; **d** Neutrophils. **e** Representative HE staining of lung tissues showed the large infiltration of inflammatory cells in OVA-induced mice; Representative PAS staining showed widely globet cell hyperplasia in OVA-induced mice compared with PBS-induced mice. Sirus red staining showed large amount of type I, III collagen deposition in OVA-induced mice. (I: red or orange; III: green; IV: light yellow) *P < 0.05 OVA vs. PBS; ***P < 0.001 OVA vs. PBS

Eosinophils are critical for the development of airway remodeling [28]. We showed that chronic exposure to OVA induced a robust airway inflammation, characterized by the accumulation of inflammatory cells, such as eosinophils, lymphocytes and netrophils. Elevations in the total cell numbers, and the percentages of macrophages, eosinophils, lymphocytes and neutrophils (7.56 ± 1.53 × 10^6^/ml, 28.20 ± 1.95, 35.40 ± 2.02, 26.70 ± 2.38 and 7.08 ± 0.74 %, respectively) were observed in the BALF of OVA-exposed mice compared with the values that were observed in PBS controls (3.69 ± 0.98 × 10^6^/ml, 68.88 ± 2.73, 6.25 ± 0.95, 18.12 ± 1.58 and 3.02 ± 0.63 %, respectively) (Fig 6a-d). Sirius red staining could distinguish collagen types. In our study, we interestingly found large amount of type I and III collagen deposition surrounding the peribronchioles, which directly evidenced airway remodeling existed in mice (I: orange; III: green;). Furthermore, PAS staining revealed that OVA induced goblet cell hyperplasia in the airway epithelium while PBS showed little change (Fig 6e). These results clearly indicate that OVA effectively promotes airway remodeling in mice.

### HuR, TGF-β1 and α-SMA were upregulated in chronic OVA-exposed asthmatic mice airways

**Fig. 7:**
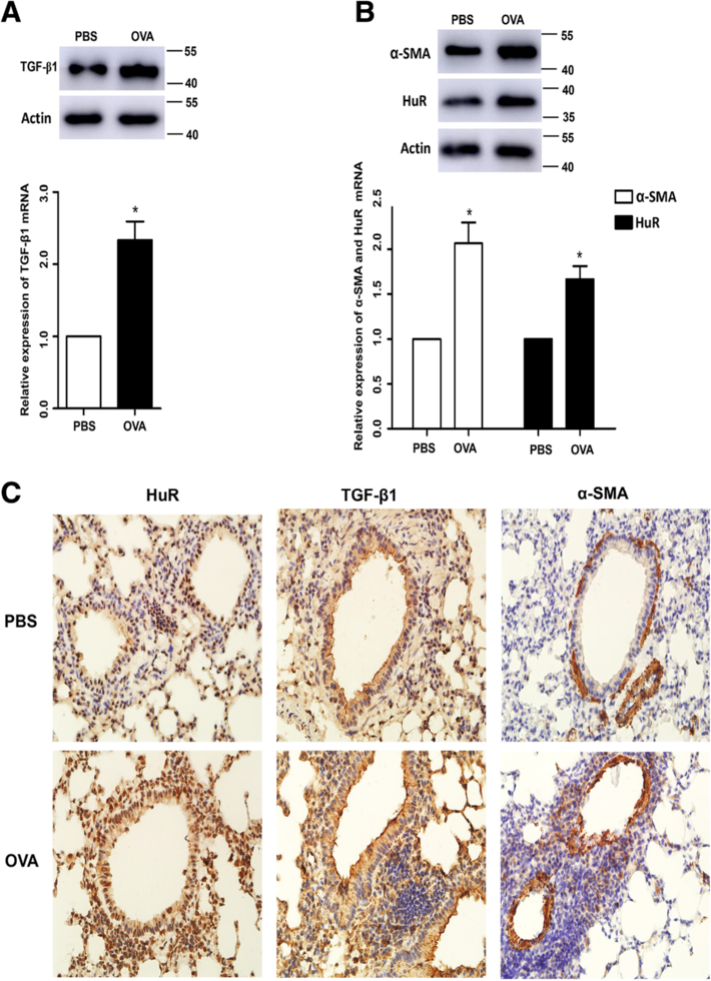
HuR, TGF-β1 and α-SMA were upregulated in chronic OVA-exposed asthmatic mice airways. **a**–**b** Representative protein bands of TGF-β1, HuR and α-SMA were determined by western blotting. mRNA levels were measured by RT-PCR in lung tissues of mice. β-actin in western blotting and GAPDH in RT-PCR were the internal control. *P < 0.05 OVA vs. PBS; **c** Representative HuR, TGF-β1 and α-SMA expression were determined by immunohistochemistry; the positive area was calculated as positive area/total bronchiole area in similar size bronchia from each group

To further understand the HuR/TGF-β1 feedback in vivo, we found that chronic OVA-challenged mice also highly expressed HuR, TGF-β1 and α-SMA in mRNA levels and protein levels of lung tissues (Fig 7a&b). As shown in Fig 7c, the results of immunohistochemistry showed a significant increase of three proteins in OVA-induced mice, which was well consistent with the results of western blotting. Overall, these data illustrated that OVA-induced airway remodeling is associated with the high expression of HuR, TGF-β1 and α-SMA.

## Discussion

In vitro and in vivo, the present study found that the effects of HuR and TGF-β1 are inter-dependent, and the balance among these interactions plays an important role in mediating airway remodeling. Here, we uncovered a HuR/TGF-β1 feedback that modulates α-SMA and Col-I expression in ASM cells or asthmatic animal mice.

Asthma is a common respiratory disease and the occurrence of which might be associated with the over-expression of asthma-associated genes. Human antigen R (HuR), an ubiquitously expressed RNA-binding protein, is known to regulate the turnover of mRNA for inflammatory genes or cell cycle proteins by binding to adenylate-uridylate-rich elements and related motifs presented in the 3′untranslated region (UTR) of mRNAs. Our previous studies demonstrated that HuR could enhance cyclinD1 mRNA stability by recognizing the 3′UTR, which controlled the proliferation of ASM cells. Airway remodeling, a key feature of asthma, is characterized by matrix deposition and enhanced smooth muscle mass in the airways. For years, multiple intracellular signal pathways have been shown to be involved. p38 mitogen-activated protein kinase (MAPK) signaling has been shown to be a center to the TGF-β1-induced collagen and fibronectin expression in systemic sclerosis (SSc) fibroblasts [29]. As TGF-β1 induces fibroblasts to synthesize and ECM contract, this cytokine has long been believed to be a central mediator of the fibrotic response [30]. In light of the analysis above, whether HuR is involved in matrix deposition and how it mediates this event in cultured ASM cells is the focus of our study, and we set to explore the interaction between HuR and TGF-β1 with the goal of defining a new role for HuR in asthma.

Firstly, we found that PDGF stimulation significantly elevated the expression of TGF-β1, α-SMA and Col-I both in the mRNA and protein levels at a time-dependent manner. Furthermore, the result of immunofluorescence of HuR and TGF-β1 were well consistent with western blotting and PCR analysis. These data proved that PDGF stimulation significantly elevated the expression of TGF-β1, α-SMA and Col-I. Secondly, RNA-interference decreased the expression of HuR to a very low level. Suppression of HuR resulted in higher proportion of apoptosis in the HuRsi group than in the Consi group and the contents of TGF-β1, Col-I and α-SMA either in intracelluar or in the cultured medium were reduced in HuRsi group. Furthermore, we detected that the half-life of TGF-β1 mRNA was also much shorter compared with the control, which was similar to some reported studies demonstrating that HuR could regulate target mRNA expression by prolonging or shortening the mRNA half-life [31–33]. Thirdly, to further explore the association between HuR and TGF-β1, TGF-β1 was inhibited by specific small interference RNA. We interestingly found that the knockdown of TGF-β1 also alleviated HuR and Col-I expression in western blotting and extra addition of TGF-β1 could partially recover HuR and Col-I expression in ASM cells, which gives us confidence that a HuR/TGF-β1 may exist to modulate airway remodeling. At last, OVA-driven mice showed widely infiltration of inflammatory cells around the bronchiole compared with PBS-induced mice. Sirius red staining distinguished collagen type I and III deposition surrounding the bronchiole especially in the OVA-induced mice. Immunohistochemistry plus western blotting and RT-PCR showed higher levels of HuR, TGF-β1 and α-SMA in the OVA -induced mice. Thus we hypothesized that a HuR/TGF-β1 feedback is involved in airway remodeling and targeting them may have considerable potential for the control of asthma.

B Wightman [34] ever demonstrated that stretch augmented TGF-β1 expression through the enhanced activation of the promoter,which indicating that targeting the posttranscriptional regulatory modules in the TGF-β1 signaling pathway is a promising approach to control airway remodeling by ASM cells. Danna Bai [27] used RNA-IP to verify that HuR could recognize the 3′UTR of TGF-β1 in cardiac fibroblasts. Their study established that a TGF-β1/HuR feedback circuit regulated the fibrogenic response in fibroblasts. This mechanism should also exist in other organisms on the basis that both HuR and the ARE in the TGF-β1 3′UTR are conserved among various species. Increased TGF-β1 would induce a cascade of the fibrogenic response in both the canonical Smad-dependent signaling pathway and non-Smad pathways by inducing the expression of collagen, fibronectin and other ECM molecules [4]. However, we have only tested a limited indicator of remodeling, Col-I and α-SMA. Numerous publications have confirmed that proliferative cytokines [35] and proinflammatory factors [36] are also involved in airway remodeling. It is therefore an important challenge to test other potential targets that are involved in controlling the process of airway remodeling.

Previous studies have shown that the actions of TGF-β1 on fibroblast proliferation are complex. The proliferation of mink lung fibroblasts is stimulated by low concentrations of TGF-β1 (5–10 ng) but inhibited by higher concentrations [37]. Treatment of ASM cells with TGF-β1 (4 ng/ml) resulted in a dramatic increase in HuR and Col-I expression compared with the untreated controls or PDGF alone. Our present study established a HuR/TGF-β1 feedback that might regulate the Col-I expression in ASM cells, and suggested that targeting this pathway has considerable potential for controlling the deposition of Col-I in ASM cells. However, the mechanism by which TGF-β1 regulates Col-I and α-SMA expression is still unclear. In mesangial cells, TGF-β1 induction of the type I collagen promoter required the RAS/MEK/ERK MAPK cascade, and in dermal fibroblasts, this response required p38 [38]. Induction of fibronectin by TGF-β1 in fibroblasts was Smad-independent but required the JNK MAPK cascade and c-jun [39]. Our next study will further explore the novel pathway between TGF-β1, α-SMA and Col-I in ASM cells.

More than 300 million individuals worldwide are suffered from asthma, and by 2025 the prevalence is predicted to increase by 100 million [40]. At present, inhaled corticosteroids are the standard therapy for persistent asthma; however, the antioxidant effects of corticosteroids are unsatisfied. Furthermore, this treatment is hindered when steroid dependence or steroid resistance occurs [41]. Therefore, the development of novel and efficient therapeutic strategies is of great significance in the control of asthma. We have described a HuR/TGF-β1 feedback that mediates airway remodeling in vivo and in vitro. Although experimental studies have adequately demonstrated that blocking these signaling pathways, especially TGF-β1, could effectively decrease airway remodeling, no such chemical inhibitors are clinically available at the present time. Interventions that target TGF-β1 are limited to acute situations, such as immediately after surgery, that would require application of the anti-fibrotic for a limited period. Such strategies might not be appropriate for the treatment of chronic fibrotic diseases that develop over many years, on the basis of the long period of dosing necessary for such diseases.

The post-transcriptional regulation of gene expression includes mRNA transportation, procession, turnover, and mRNA translation.Targeting this pathway has considerable potential to control the deterioration of lung function due to asthma. HuR, as an upstream modulator of TGF-β1, serves to provide a novel insight for searching the precise target of asthmatic airway remodeling. Thus, the future research will focus on silencing HuR in mice to explore a new road being clinically appropriate for controlling asthma.

## Conclusion

A HuR/TGF-β1 feedback circuit was established to regulate airway remodeling in vivo and in vitro and targeting this feedback has considerable potential for the control of asthma.

## Abbreviations

ASM cells: Airway remodeling smooth muscle cells
BALF: Bronchoalveolar lavage fluid
Col-I: Collagen I
ELISA: Enzyme-linked immunosorbent assay
HRP: Horseradish peroxidase
HuR: Human antigen R
OVA: Ovalbum
PBS: Phosphate buffered saline
PDGF: Platelet derived growth factor
TGF-β1: Transforming growth factor β1
α-SMA: α-smooth muscle actins
